# Lung commensal bacteria promote lung cancer progression through NK cell-mediated immunosuppressive microenvironment

**DOI:** 10.7150/ijms.107026

**Published:** 2025-02-03

**Authors:** Haiyang Wang, Jiayi Hu, Yirou Ma, Yilimunuer Abulimiti, Yongxin Zhou

**Affiliations:** 1 Department of Laboratory Medicine, Tongji Hospital of Tongji University, School of Medicine, 389 Xincun Road, Shanghai 200065, China.; 2 Department of Thoracic and Cardiovascular Surgery, Tongji Hospital of Tongji University, School of Medicine, 389 Xincun Road, Shanghai 200065, China.

**Keywords:** Lung cancer, NK cells, Bacteria, Immune microenvironment, TIGIT

## Abstract

Symbiotic microbiota pervades the majority of the human body's organs and tissues, functioning as crucial regulators of both health maintenance and disease progression. Pertinently, lung adenocarcinoma has been indisputably linked to chronic inflammation. However, the precipitators that instigate such inflammation, along with the particular immune mediators involved, remain enigmatic and warrant extensive exploration. This research revealed a significant variance exists in the commensal bacteria between lung cancer tissues and their normal counterparts. This holds true for both clinical patients and mice, where both the diversity and abundance of bacteria in tumor tissues significantly surpass those in normal tissues. It has been demonstrated that disturbances in pulmonary commensal bacteria can stimulate the proliferation of tumor cells. Mechanistically, we suggest that lung bacteria may promote the expression of the NK cell immunosuppressive molecule TIGIT along with the secretion of IL-2 and IFN-γ. This consequently mediates alterations in the immunosuppressive microenvironment, thereby fostering tumor proliferation.

## 1. Introduction

Lung cancer, which is the predominant cause of cancer-related fatalities across the globe, accounts for a mortality rate higher than the aggregate death toll of various other cancers such as breast, colon, prostate, and kidney 1. The lung, having the largest surface area in the human body and responsible for gas exchange, is inevitably exposed to various environmental microorganisms. Though the impact of the lung microbiome on lung cancer remains ambiguous, observational studies have alluded to a correlation between repeated exposure to antibiotics and a heightened risk of developing lung cancer 2, 3. There is an expanding body of evidence suggesting the lung microbiome's potential involvement in cancer initiation. Lung cancer is often concomitant with conditions including COPD, HIV, and Chlamydia infections, all of which are frequently associated with persistent lung infections 4-6. In experimentally challenged germ-free rats, lung cancer development is less frequent than in conventional control rats 7. Studies have illustrated that the prolonged administration of LPS in mice precipitates the emergence of lung tumorigenesis 8. Additionally, in a mouse model, the propagation of commensal bacteria influenced by antibiotics impacts the response of γδT17 cells, thereby facilitating aggressive metastatic development in pulmonary tumors, hence underscoring the significant role of microbiota 9. All of these studies indicated that lung microbiota may play an essential role in pathogenesis of lung cancer.

Current observations propose that a perturbed lung or lower airway microbiota can potentially impact lung carcinogenesis via various mechanisms. These mechanisms include inciting host inflammatory pathways, releasing bacterial toxins that destabilize host genomic stability, and the emission of microbial metabolites that promote cancer 5. Distinct lung microbiota identified in the lower airways have been shown to impact the host immune phenotype and many signal pathways 10, 11. For instance, Jun-Chieh *et al.* found that enrichment of *Veillonella* in lower airway and lung is associated with increased infiltration with inflammatory cells (Th17 cells) and upregulation of the ERK (extracellular signal-regulated kinase)/PI3K (phosphoinositide 3-kinase) pathway 12. Importantly, elevation of the PI3K pathway, demonstrated in prior studies, is an initial pathogenic incident in non-small cell lung carcinoma. It modulates cell proliferation, survival, differentiation, and invasion 13. The tumor suppressor gene TP53 is the most commonly mutated gene in lung cancer 14, with certain missense mutations showing gain of oncogenic function 14. Although it has been reported that commensal bacteria may mediate TP53 mutations 15, the specific intrinsic relationship and mechanism have not been elucidated. In addition, the loss of p53 in enterocytes in murine models impairs the epithelial barrier and allows infiltration of bacteria resulting in NF-κB signaling, which is required for tumor progression 16.

In this study, we developed a spontaneous mouse model of lung adenocarcinoma to demonstrate through a sequence of *in vivo* and *in vitro* experiments that bacterial diversity in tumor tissue was elevated. We further confirmed that these bacteria might foster tumor progression by adjusting the inhibitory immune microenvironment. This study lays the groundwork for mechanistic explorations of lung adenocarcinoma evolution driven by lung resident bacteria.

## 2. Methods

### 2.1 Ethics

Tumor samples were obtained from 19 patients with invasive lung adenocarcinoma who underwent surgical treatment at Tongji Hospital of Tongji University between 2022 to 2023. All samples were collected from patients with informed consent, and all related procedures were performed with the approval of the internal review and ethics boards of the indicated hospitals. All animal experiments of this study were carried out in accordance with the recommendations of the Guide for the Care and Use of Laboratory Animals of the National Institutes of Health. The protocol was approved by the Animal Research Ethics Board of Tongji Hospital of Tongji University (Approve number: 20220401-DW-077).

### 2.2 Animals

In this model, Kras^G12D^ mice were constructed as follows. In general, targeting vector was designed to introduce a G12D mutation to exon 1 of the gene and place a pgk-neomycin resistance cassette in intron 1. The construct was electroporated into 129S4/SvJae-derived J1 embryonic stem (ES) cells. Mice carrying the pgk-neo cassette flanked by an insertional duplication of the G12D exon 1 mutation were selected for further study (K-rasLA2 allele). In vivo recombination/excision events between the two mutant exons occur in all carrier animals, excising the pgk-neo cassette and leaving a single G12D mutation to confer the oncogenic phenotype, namely lung adenoma. This spontaneous oncogene activation closely recapitulates that observed in human cancers. Mice were bred and maintained in a specific-pathogen-free (SPF) or germ-free (GF) environment at the Center of Experimental Animal Sciences in the Tongji Hospital of Tongji University. Food and water were provided *ad libitum*.

### 2.3 *In vitro* cell co-culture

LLC or A549 cells (ATCC, USA) were uniformly distributed within the well plate. Once the cells had fully adhered and extended, they were co-incubated with the heat-inactivated bronchoalveolar lavage fluid (BALF) derived from mice. BALF after centrifugation at 15000 rcf for 30 mins was resuspended in 100 μL PBS, and added to the medium for co-culturing 72 h. The cells were then washed thrice with PBS before being harvested using a cell scraper. NK92 is a cell line grown in suspension. The ratio of tumor cells to NK cells needed to be accurately counted before co-culture was performed at 1:10. After the culture process ended, the NK cells present in the medium were isolated and subjected to a series of three PBS washes.

Tumor cells were cultured in high-glucose DMEM supplemented with 10% fetal bovine serum, maintained under conditions of 5% CO_2_ at 37℃. As for the NK92 cell line, it necessitated a specialized medium, enriched with 10% horse serum and 10ug/μL IL-2, and kept in a 5% CO_2_ environment at 37℃.

### 2.4 Bronchoalveolar lavage fluid collection

1 mL sterile PBS was injected through the trachea of mice into the lungs using a disposable sterile indwelling needle. The PBS was then gently withdrawn with the syringe and reinjected into the lungs. This rinsing-and-recycling process was iterated three times to maximize the collection of lung-resident cells, alveolar microbiota, and shed metabolites. The BALF was centrifuged at 500 rcf for 5 mins, the supernatant was discarded, and the cells were resuspended and washed twice more with PBS. BALF after centrifugation at 15000 rcf for 30 mins was resuspended in 100 μL PBS, and subjected to high-pressure steam sterilization for 15 minutes.

### 2.5 Flow cytometry

To quantify the cytokine and immune cell infiltration in the lung of mice, four flow cytometry panels were established to quantify cytokines, the relative proportion of T cells and NK cells, and NK cytotoxicity, respectively. The BALF used for cytokine measurement was obtained directly from 50 μL of the supernatant for flow cytometry. Samples used for immune cell infiltration analysis were washed twice with PBS, and immune cells were isolated according to the instructions of Percoll isolation and washed twice with PBS. Following blocking FcgRIII/II with an anti-CD16/CD32 mAb (eBioscience), single cell suspensions were stained with the following antibodies: CD45, CD3, CD4, CD8, CD19, NK1.1, TIGIT. Flow cytometry was performed on the 2L B-R Fluorochrome (Cytek), and data were analyzed by the FlowJo software (Treestar).

### 2.6 Tissue sections and staining

Mice were euthanized after administering an intraperitoneal injection of 50 μg/μL pentobarbital sodium (100 μL total), and endotracheal intubation was performed with an indwelling needle to perfuse the alveoli. The intact lungs were removed from the mice and fixed in 4% paraformaldehyde for a minimum of 24 h before undergoing dehydration paraffin embedding, and section staining. Hematoxylin and eosin stain (H&E stain) was conducted following a standard method (KI Histology Facility). IHC staining of Ki67 was carried out on 5 μm unstained sections after antigen retrieval with citrate buffer or proteinase-K (Sigma) respectively. Digitally scanned images of H&E or Ki67-stained slides were generated using the NanoZoomer Digital Pathology at 20X magnification and analyzed with the Image J software.

### 2.7 DNA extraction and amplification and sequencing of 16S rRNA gene

Total DNA from bacteria was extracted under the instruction of QIAmp DNA Mini Kit (Qiagen, Hilden, Germany). Subsequently, the concentration was tested using an ultra-microspectrophotometer (Thermo, Nanodrop OneC, USA). The primers of the full-length 16S rRNA gene were as follows: 27F (AGRGTTTGATYNTGGCTCAG) and 1492R (TASGGHTACCTTGTTASGACTT), synthesized by Sangon Biotech, Co., Ltd. The PCR reaction conditions were set as follows: 95℃ for 5 min, 95℃ for 30 s, 50℃ for 30 s, 72℃ for 1 min, with 30 cycles in total in a reaction volume of 10 μL. PCR products were then purified, quantified and homogenized to construct a sequencing library (SMRT Bell). Pair-end raw sequencing data were processed with QIIME version 1.8.0. Demultiplexing, adaptor trimming, and read merging were performed by the default QIIME pipeline. Chimeric sequences of merged reads were detected and removed by USEARCH (version 11.0.667) in QIIME (Edgar 2010). After that, the species composition of each sample can be revealed through filtering and clustering or denoising of Circular Consensus Sequencing (CCS) sequences, as well as species annotation and abundance analysis.

### 2.6 Species notes and taxonomic analysis

The clean sequences were subsequently clustered into operational taxonomic units (OTUs) at 97% similarity level by USEARCH. Representative sequences from each OTU cluster were aligned and annotated by the Ribosomal Database Project (RDP) classifier against the SILVA (http://www.arb-silva.de) database, with a minimum confidence of 0.8. Mitochondrial and chloroplast reads were then removed, and an OTU table was resampled to obtain an equal number of read numbers per sample.

Species abundance and diversity were determined by alpha diversity including the Shannon, Simpson, Chao1 and ACE indices. Principal coordinates analysis (PCA) was based on the OTU abundance of sequenced samples, and Bray-Curtis dissimilarities were computed using the “vegan” package in R. Variation analysis in PCA was calculated by PERMANOVA with 999 permutations.

Metastats software was employed to compare the species abundance between the groups and the calculated p-value (p≤0.05) was further corrected to obtain the q-value. The significance test between the groups was respectively performed at various taxonomic levels, including phylum, class, order, family, genus, and species.

### 2.7 Statistical analysis

MOTHUR was used to calculate the rarefaction curves, alpha diversity and richness indices and UniFrac distance. Student's t-test and Mann-Whitney U test were used to test the difference of indices of diversity and richness of salivary microbiome (version 19.0, SPSS, Chicago, IL, USA). Differences among individual taxa and sample characteristics in the two groups were investigated with nonparametric Kruskal-Wallis testing, followed by post-hoc between group comparisons with a Mann-Whitney U test. Correlations between the clinical phenotype and the characteristics of samples were tested by Spearman and multiple linear regression. The level of statistical significance was p=0.05.

GraphPad Prism 7 was utilized for statistical analysis of the histology/IHC/16S quantification and flow cytometry data. P-values from unpaired two-tailed student's t-tests were used for comparisons between two groups and one-way ANOVA with Bonferroni's post hoc test was used for multiple comparisons.

## 3. Results

### 3.1 The commensal bacteria in lung cancer tissues displayed a higher level of diversity

**Figure 1:**
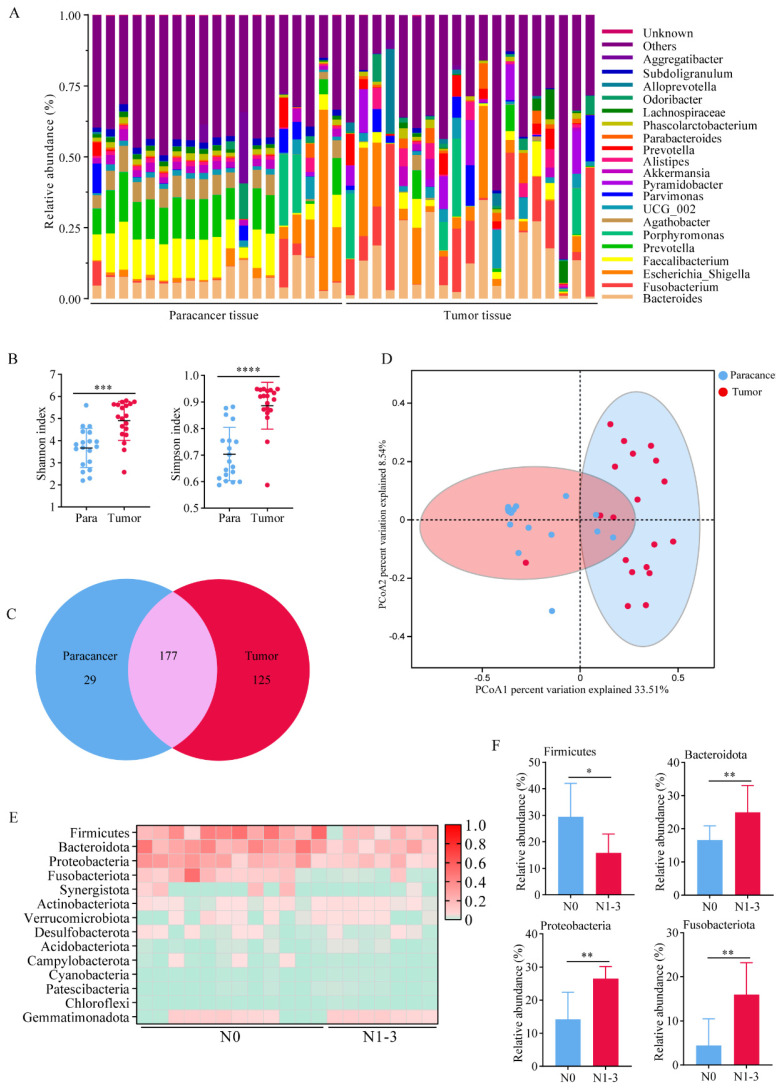
The commensal bacteria in lung cancer tissues showed higher diversity. A Bacteria abundance histogram of paracancer tissue and tumor tissue. B Alpha diversity of the microbiota was higher in tumor tissues compared to paracancer tissues. C Venn diagram showed shared and unique bacteria of tumor and paracancerous tissues. D PCoA analysis showed a difference in 16S rRNA between the paracancer tissue and tumor tissue. E A heat map illustrated the differences in the composition between N0 and N1-3 of lung cancer patients. F Bacteria with significant differences in abundance at the genus level.

We performed 16S rRNA sequencing on cancerous and adjacent tissues collected from 19 clinical lung adenocarcinoma patients. The analysis of the relative abundance of bacteria at the genus level revealed that the bacterial flora in tumor tissues showed a higher level of heterogeneity compared to that in the paracancer tissues (Figure 1A). Consistent to some extent with relative abundance analysis, alpha diversity of the microbiota including Shannon index and Simpson index were higher in tumor tissues compared to paracancer tissues, and the statistical difference was found (Figure 1B). As expected, we found 177 shared genera in both tumor and paracancer tissues of lung cancer patients. 125 genera were unique to tumor, including *Fusobacterium* and *Faecalibacterium*, which were potential biomarkers in tumor tissues (Figure 1C). 29 genera were unique to paracancer. Moreover, PCoA using Bray-Curtis distance also showed a difference in 16S rRNA between the paracancer tissue and tumor tissue. (Figure 1D). The results indicated significant differences in the symbiotic bacteria between tumor tissues and adjacent tissues. These differences could potentially be a critical factor contributing to the malignant progression of lung adenocarcinoma.

To explore the relationship between lung cancer progression and bacteria. We divided 19 patients into stage N0 (n=12) and stage N1-3 (n=7), according to TNM staging of lung cancer. Patients in stage N0 or N1-3 showed no differences in age, gender, BMI, smoking history, or pathology (Table 1). At the genus level, we created a heat map to illustrate the differences of relative abundance of bacteria (Figure 1E). We found that *Bacteroidota*, *Proteobacteria* and *Fusobacteriota* showed relatively higher abundance in both groups (Figure 1F). These results suggested that the structure and composition of lung bacteria may change with the progression of tumor, and these changes may in turn promote the malignant proliferation of tumor cells.

### 3.2 Commensal bacteria of primary lung cancer tissues in mice showed higher heterogeneity

**Figure 2:**
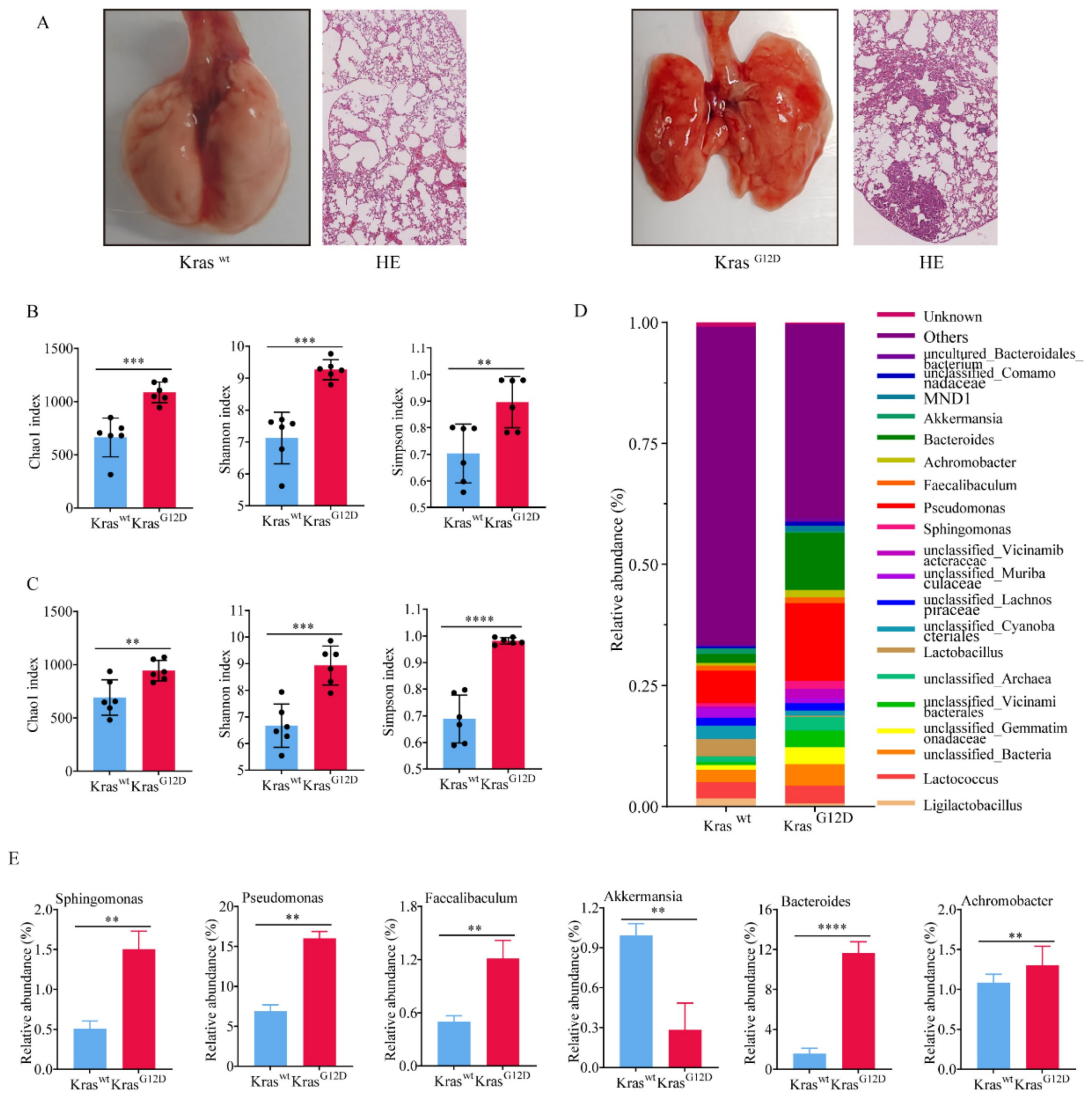
Commensal bacteria of primary lung cancer tissues in mice showed higher heterogeneity. A Lung cancer model mice constructed based on the Kras^G12D^ mutation. B-C Symbiotic bacteria alpha diversity of tumor tissue (B) and BALF (C). D Bacteria abundance histogram at the phylum classification level of tumor tissue. E Bacteria with significant differences of Kras^G12D^ and Kras^wt^ mice.

To investigate the impact of lung microbiota on the onset and progression of lung adenocarcinoma, we successfully engineered a mouse model of lung cancer exhibiting a Kras^G12D^ mutation (Figure 2A). Both newborn Kras^G12D^ mutation and wild type mice (Kras^wt^) were kept in specific pathogen free (SPF) environment for a duration of ten weeks. Despite residing in identical environments, there was a significant difference in the diversity and abundance of symbiotic bacteria. We discovered that the alpha diversity and abundance (Figure 2B) of symbiotic bacteria in the tumor tissue of Kras^G12D^ mice were notably greater than in Kras^wt^ mice. Similarly, we extracted BALF from both groups, and the results paralleled that of the tissues; the diversity of lung symbiotic bacteria in the Kras^G12D^ mice was significantly elevated compared to the Kras^wt^ mice (Figure 2C). This suggested that both intra-tumoral and extra-tumoral symbiotic bacteria exhibit higher heterogeneity than those in the control group.

Furthermore, we founded that at the phylum classification level (Figure 2D), five bacterial phyla in the tumor tissue of Kras^G12D^ mice were notably higher than in the Kras^wt^ mice (Figure 2E), with only one bacterial phylum being significantly lower in comparison to the Kras^wt^ mice (Figure 2E). This insinuated that the irregular composition of these bacteria could contribute to the progression of lung cancer. Indeed, collectively, our findings revealed a higher diversity and abundance of bacteria in tumor tissue compared to normal lung tissues. Nonetheless, the implications of these discrepancies on the progression of lung cancer require additional confirmation.

### 3.3 Commensal microbiota promotes tumor growth in spontaneous lung adenocarcinoma

**Figure 3:**
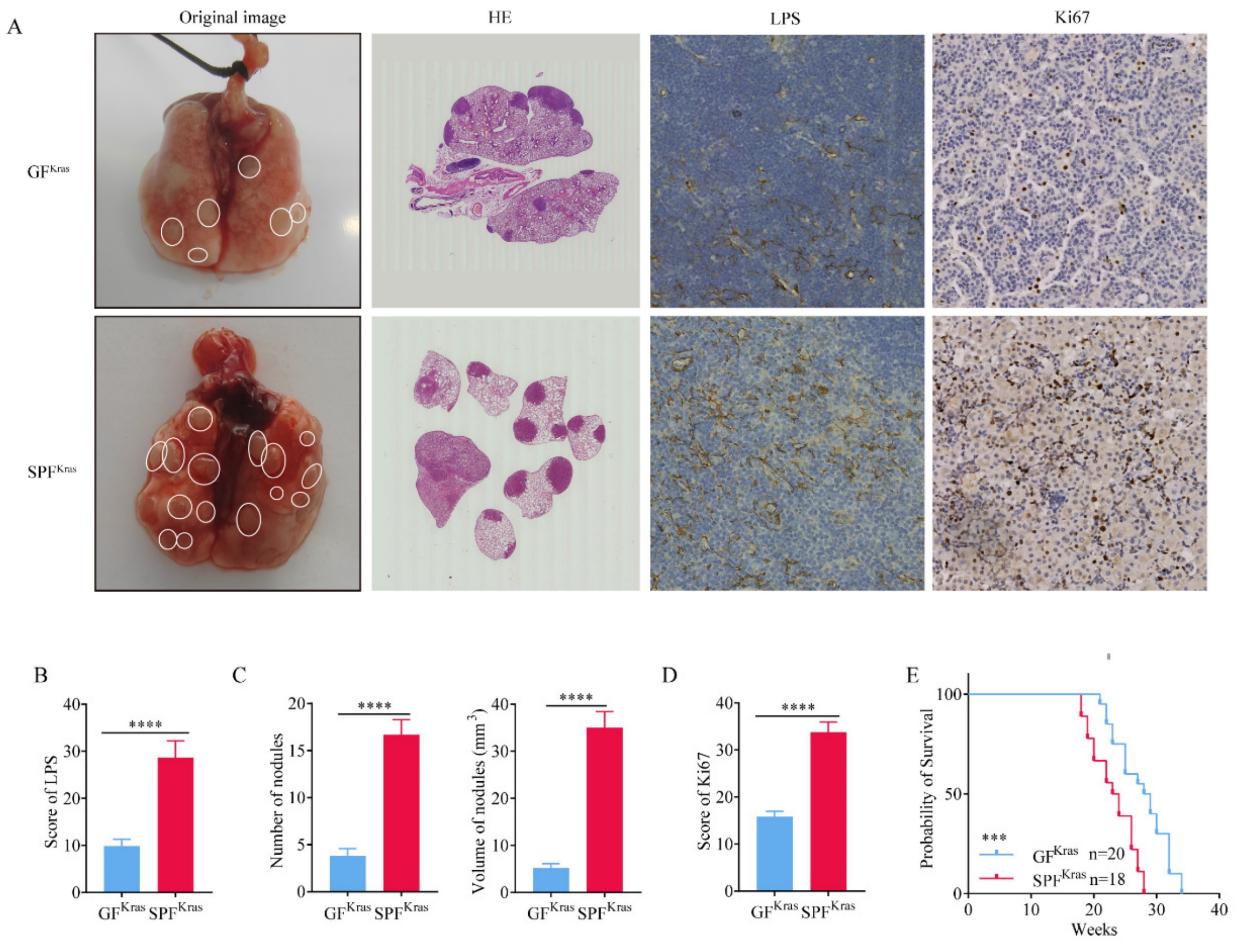
Commensal microbiota promotes tumor growth in spontaneous lung adenocarcinoma. A-B H&E stain was performed and immunohistochemical tests was performed to quantify the abundance of bacteria and positive rate of Ki67. C Number of lung nodules and the volume of tumors in SPF^Kras^ mice greatly exceeded those in GF^Kras^ mice. D The positive rate of Ki67 in SPF^Kras^ mice were significantly higher than those of GF^Kras^ mice. E The survival rate of GF^Kras^ mice was much higher than that of SPF^Kras^ mice.

Newborn Kras^G12D^ mice were fed in germ-free (GF) and SPF environments for 16 weeks, respectively. After whole lung dissection of mice fed in GF (GF^Kras^) and SPF (SPF^Kras^), we first performed immunohistochemical tests to quantify the abundance of bacteria in lung tissues, using anti-LPS antibodies. As anticipated, relative abundance of bacteria in SPF^Kras^ was significantly higher than that in GF^Kras^ mice (Figure 3A-B), suggesting that the lungs allow bacteria to reside by exchanging them with bacteria in the environment. Subsequently, we discovered that both the number of lung nodules and the volume of nodules in SPF^Kras^ mice greatly exceeded those in GF^Kras^ mice (Figure 3C). These findings implied that the disruption of the lung's bacterial structure could potentially accelerate the proliferation of lung adenocarcinoma in mice. In addition, through the analysis of the positive rate of tumor proliferation index Ki67, it was found that the positive rate of Ki67 in SPF^Kras^ mice were significantly higher than those of GF^Kras^ mice (Figure 3A, 3D), further indicating that bacteria expansion can promote the proliferation of tumor cells. Finally, by calculating the survival curve of the two groups of mice, we learned that the survival rate of GF^Kras^ mice was much higher than that of SPF^Kras^ mice (Figure 3E), highlighting once more the potential of disrupted lung flora to advance the malignant progression of lung adenocarcinoma in mice and reduce the survival rate of mice.

**Figure 4:**
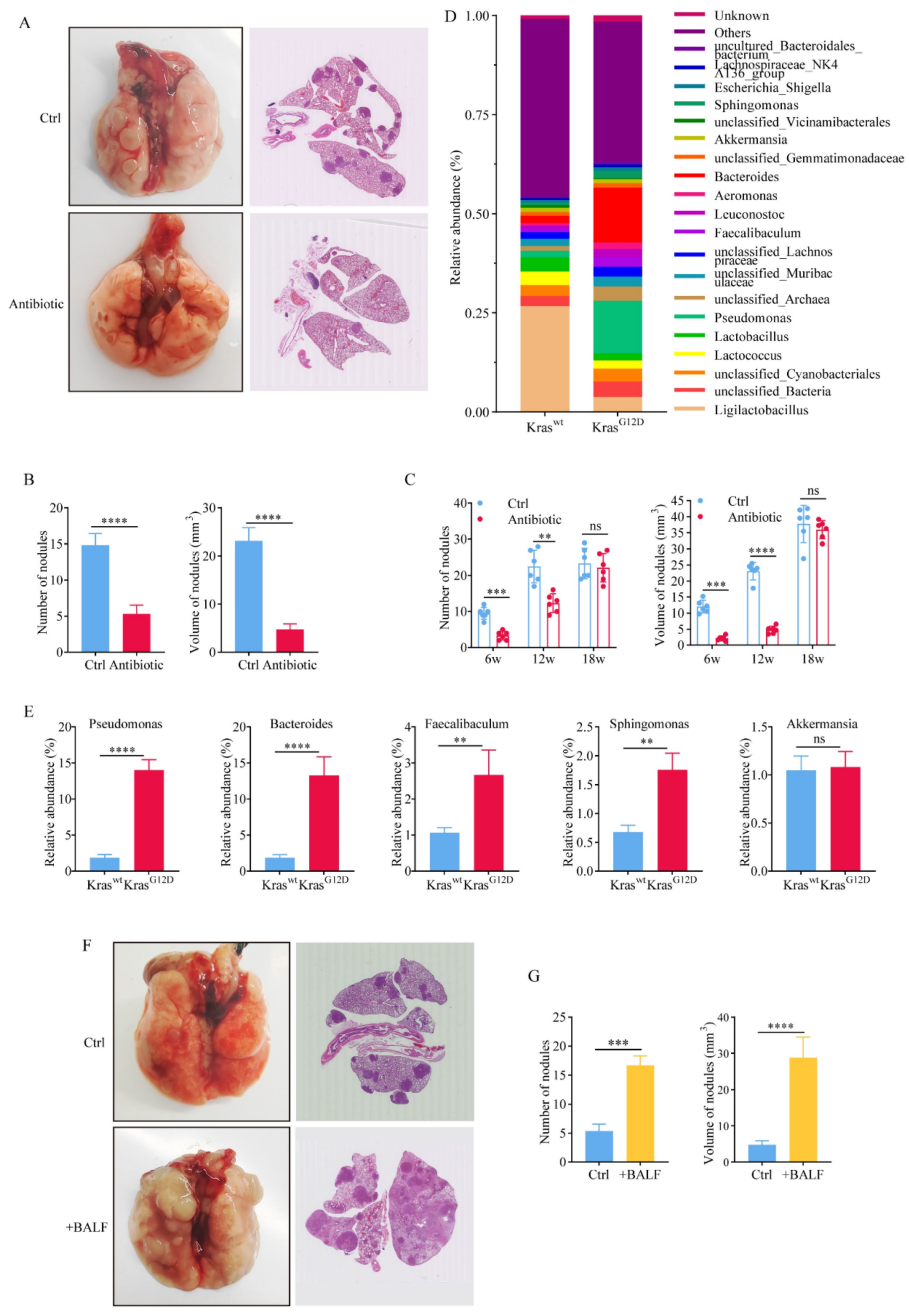
Commensal microbiota promotes tumor growth in spontaneous lung adenocarcinoma. A H&E stain after administrating antibiotics to mice fed in GF environment for 15 weeks. B The number and volume of lung nodules in the antibiotic-treated group were much smaller than those in the control group. C Continuous antibiotic treatment for 6 and 12 weeks significantly inhibited the proliferation of lung cancer. D Relative abundance of bacteria in Kras^wt^ and Kras^G12D^ BALF. E The relative abundance of bacteria at the genus classification levels of BALF. F +BALF significantly promoted the lung cancer. G The number and volume of tumors in +BALF mice were significantly higher than those in the control group.

In order to demonstrate that dysbacteriosis can affect the proliferation of lung cancer, we administrated antibiotics (vancomycin 0.5g/L and polymyxin 1g/L) to mice fed in GF environment for 15 weeks (Figure 4A), and found that the number and volume (Figure 4B) of lung nodules in the antibiotic-treated group were much smaller than those in the control group (Figure 4A, 4B). These results indicated that antibiotic treatment could inhibit the proliferation of lung cancer by inhibiting the abnormal changes of lung flora. We then treated the mice in a sterile environment with antibiotics at different times, and found that continuous antibiotic treatment for 6 and 12 weeks could significantly inhibit the proliferation of lung cancer (Figure 4C), while continuous antibiotic treatment for 18 weeks showed no significant change in the number of lung nodules in the mice (Figure 4C). This outcome might be attributed to the emergence of bacterial drug resistance due to the extended use of antibiotics. Alternatively, it could also be a result of the multitude and complexity of factors influencing the malignant growth of lung cancer, particularly as the disease progresses to the advanced stages. What we can assert with certainty is that antibiotics do not directly impede tumor proliferation. We speculated that antibiotics thwart the factors within the tumor's microbial community that are conducive to cancer propagation.

Symbiotic bacteria in mouse BALF can objectively reflect the composition of mouse lung flora. Therefore, we analyzed the relative abundance of bacteria in Kras^wt^ and Kras^G12D^ BALF (Figure 4D). The bacterial composition of BALF was similar to that of lung tissue. Notably, Kras^G12D^ mice exhibited significantly higher levels of certain bacterial genera such as *Pseudomonas*, *Bacteroides*, *Faecalibaculum*, and *Sphingomonas* compared to Kras^wt^ mice (Figure 4E). In order to prove that the abnormal structure of lung flora can promote the proliferation of lung cancer, we transfused the precipitate of BALF from mice with advanced lung cancer back into mice with early-stage tumor. After feeding in a germ-free environment for 15 weeks, it was found that compared with the control group that received PBS, the proliferation of lung cancer was significantly promoted in mice that received BALF back (Figure 4F). The number and volume of tumors in +BALF mice were significantly higher than those in the control group (Figure 4G). This once again proved that the abnormal structure of lung flora can promote the proliferation of tumors.

### 3.4 Microbiota promotes progression of lung cancer via TME

To investigate how the symbiotic bacteria promote lung cancer proliferation, BALF from Kras^G12D^ mice fed in SPF were ultracentrifuged, the precipitate was thermally inactivated, and then co-cultured with LLC cells. The proliferation ability of LLC cells was observed by CCK8 assay, but no significant difference was found between SPF^Kras^ and control (Figure S1A). Subsequently, we verified the proliferation and migration capacity of LLC cells under different treatments by western blot analysis (Figure S1B), which once again proved that the malignant proliferation capacity of LLC cells was not changed by BALF from Kras^G12D^ mice. Meanwhile, apoptosis experiment results based on flow cytometry showed that there was no significant difference in apoptosis between the two groups of LLC cells (Figure S1C). Taken together, we hypothesized that pulmonary symbiotic bacteria or their metabolites may not be able to act on tumor cells and promote tumor proliferation in a direct manner. Therefore, we speculated that symbiotic bacteria could be intricately influencing changes within the immune microenvironment of the tumor, thereby regulating tumor proliferation in an indirect manner.

In order to explore whether pulmonary symbiotic bacteria affect the tumor immune microenvironment (TME), we used flow cytometry to investigate the relative abundance of T cells and NK cells in BALF of SPF^Kras^ and GF^Kras^. Our results indicated that there were no significant differences in CD4^+^ T cells (Figure S1D, S1E), CD8^+^ T (Figure S1D, S1F) and NK cells (Figure S1D, S1G) between SPF^Kras^ and GF^Kras^ mice. This suggested that lung flora dysregulation might not affect the chemotaxis and recruitment of tumor-associated T cells and NK cells through a cascade reaction. Recent studies highlight the interplay between NK cells and gut microbiota, which plays a crucial role in tumor immunology by modulating tumor progression and therapeutic responses. This multifaceted relationship involves immune regulation, microbial metabolites 17, and the tumor microenvironment 18, 19. Tryptophan metabolites, derived from microbial metabolism, interact with aryl hydrocarbon receptors (AhRs) on NK cells, modulating their cytotoxicity against tumor cells 19. In addition, dysbiosis, characterized by an imbalance in gut microbiota, promotes the accumulation of myeloid-derived suppressor cells (MDSCs) and regulatory T cells (Tregs), which suppress NK cell activity 20. Lung cancer patients with high microbial diversity had a higher abundance of depleting CD8^+^ T cells and NK cell subsets in the peripherye 21. Mechanistic studies found that a high-salt diet increased intestinal permeability and the localization of intratumoral *Bifidobacterium*, which enhanced NK cell overactivation and increased their senescence phenotype and the formation of depleted NK cells, reducing antitumor immunity 22. This suggests that excessive commensal bacteria or their metabolites may provoke excessive activation even depleting of NK cells and T cells when the structure of commensal bacteria in the lung is abnormal or disordered.

**Figure 5:**
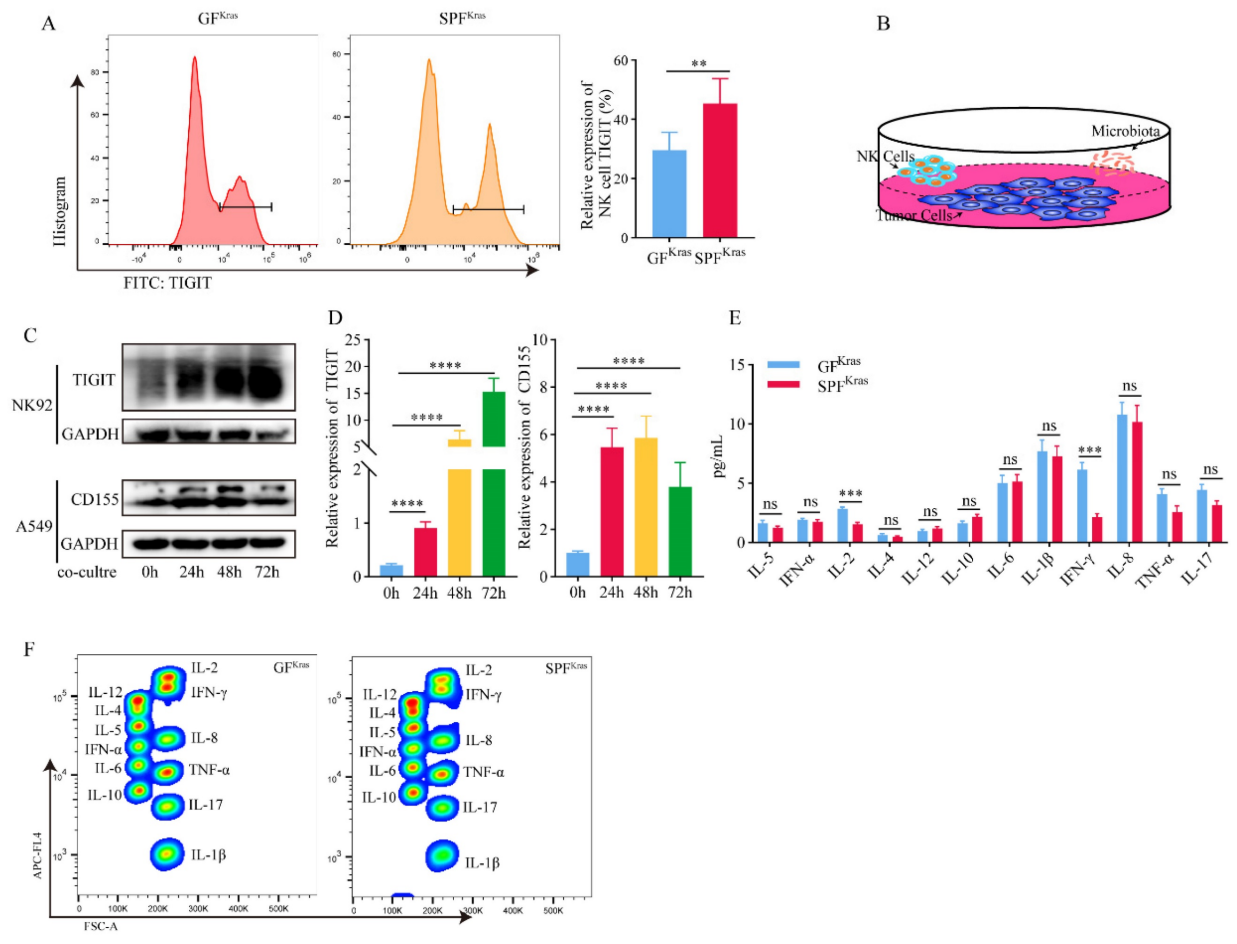
Microbiota promotes progression of lung cancer via TME. A TIGIT molecules of NK cell from SPF^Kras^ mice significantly increased. B The co-culture model diagram of NK92 cell with the A549 cell and the thermally inactivated BALF. C-D The expression of TIGIT molecule in NK92 cells and CD155 in A549 was significantly increased. E-F IL-2 and IFN-γ in SPF^Kras^ mice were significantly lower than those in GF^Kras^ mice.

After a series of screening via flow cytometry, we found a significantly increase of TIGIT molecules on NK cells from SPF^Kras^ mice (Figure 5A). TIGIT is an immunosuppressive receptor expressed on T cells and NK cells that can bind to CD155 and signal to inhibit effector T cells and NK cells, diminishing their functionality This relationship plays a key role in the depletion of lymphocytes and suppression of the immune system in various types of cancer as well as certain chronic viral infections. Based on the above ideas, we hypothesized that the continuous stimulation of NK cells by tumor antigens and bacterial metabolic antigens may lead to the gradual transformation of NK cells with killing activity into exhausted NK cells. To verify this hypothesis, we co-cultured the NK92 cell line with the A549 cell line and the thermally inactivated BALF (Figure 5B). We found that with the extension of co-culture time, the expression of TIGIT molecule in NK cells was significantly increased (Figure 5C-D). Prolonged exposure to both tumor and microbial antigens intensifies TIGIT expression in NK cells, accelerating the shift towards a depleted NK cell phenotype, which diminishes their ability to combat tumors. Subsequently, we scrutinized the expression of CD155 in A549 cells and found that although the expression of CD155 was not significantly time-dependent, it was significantly increased compared with the control group without addition of bacteria (Figure 5C-D). This may be attributed to the high immune escape function of the tumor cells. And to be under continuous stimulation of bacterial antigens, which makes them highly express CD155, leading to the depletion of NK cells.

**Figure 6:**
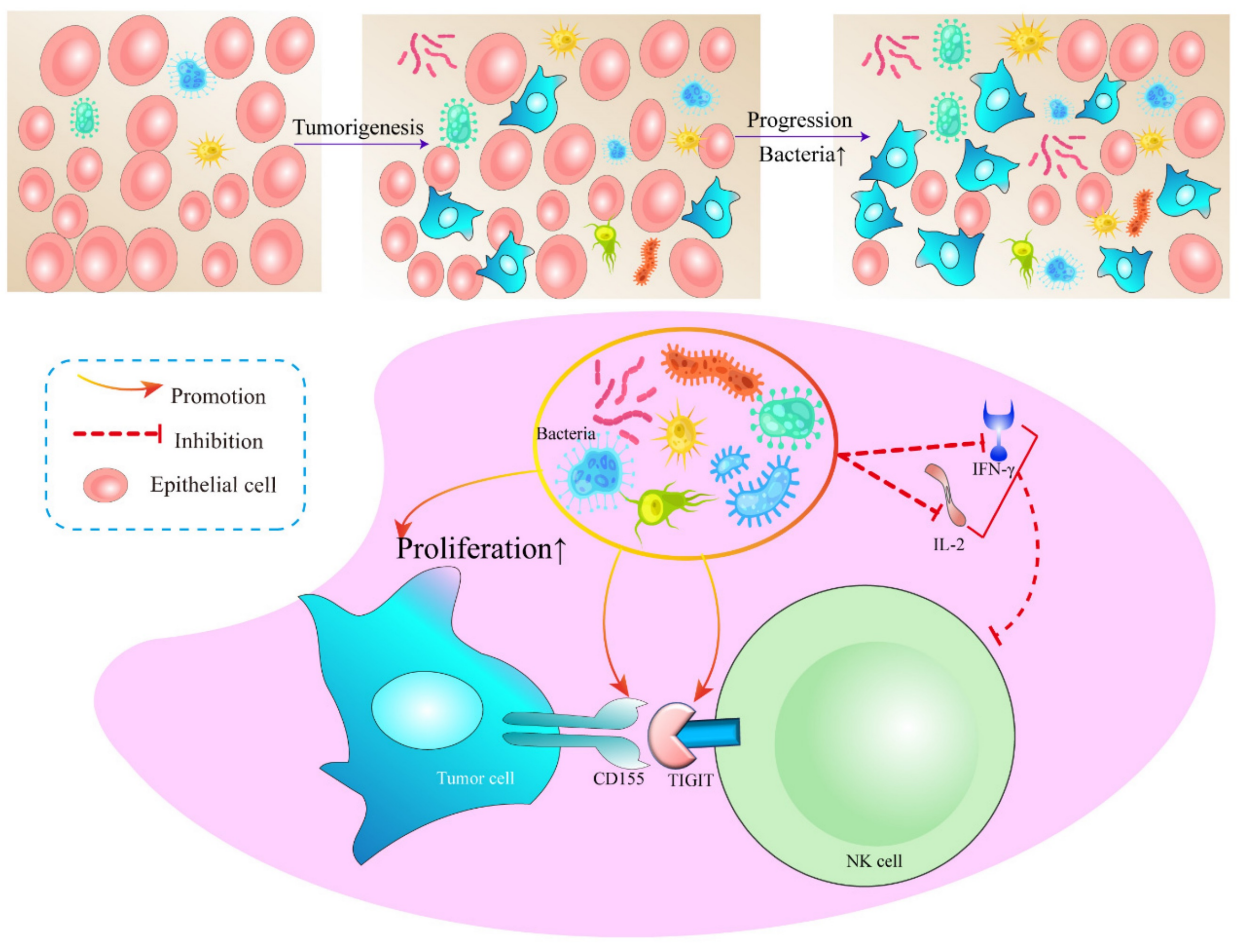
A working model in which lung symbiotic bacteria promotes lung cancer via the generate of immunosuppressive microenvironment.

In addition, we detected the main cytokines in the BALF that could reflect the immune function in the tumor microenvironment, including IL-1β, IL-2, IL-4, IL-5, IL-6, IL-8, IL-10, IL-12, IL-17, IFN-α, IFN-γ, TNF-α. It was found that IL-2 and IFN-γ in SPF^Kras^ mice were significantly lower than those in GF^Kras^ mice (Figure 5E-F). IFN-γ functions as a pivotal catalyst in the activation and propagation of both T cells and NK cells 23. Additionally, it possesses the capacity to incite glycolysis 24, 25 along with metabolic signaling pathways. These combined factors significantly contribute to instigating transformative changes within the tumor microenvironment. IL-2 has the potential to influence not only the vigor and virulence of T cells and NK cells 26, but also their chemotactic properties. These results suggested that dysregulation and an increase of microbial abundance of the lung microbiota, may contribute to the formation of an immunosuppressive microenvironment in lung cancer (Figure 6).

## 4. Discussion

Previous studies have shown that although the lung is involved in the exchange of air between the body and the external environment, the lung of a healthy person is a relatively sterile environment 27. In the past few years, a wealth of scientific literature has accentuated the link between pulmonary microbiota and lung cancer. The paradigm has now shifted from the lungs being previously considered a sterile environment to it being acknowledged as a host to a variety of microbial communities 28. Emerging evidence suggests that these resident microbes may play a significant role in the carcinogenesis of lung tissue, painting a multifaceted picture of their functional role in lung cancer development and progression 29. This suggests that, in addition to the known pulmonary bacterial pathogens, including *Streptococcus pneumoniae*, some lung engraftment symbiotic bacteria may also be involved in the occurrence and development of tumor 30. Not only that, in recent years, there are emerging lines of evidence that microbes are also integral components of the tumor tissue itself in much broader cancer types beyond colorectal cancer, such as pancreatic cancer, lung cancer, breast cancer, and others, which were originally thought to be sterile 31-33. Clinically, cohort studies have suggested that features of the tissue-resident microbiota correlate with cancer risks, pathological types, cancer prognosis, and treatment responses 31, 34. These findings suggest that both lung bacteria and intratumoral bacteria may contribute to lung cancer development, although the exact mechanisms require further investigation. This aligns with our *in vivo* results, which showed a higher diversity and abundance of bacteria reside in the tumor tissue of Kras^G12D^ mutant mice compared with Kras^wt^ mice. However, it remains unclear whether these bacteria are internal or external to the tumor cells. Additionally, we observed that the proliferation of tumor cells was enhanced when the lung microbiota was disordered. These findings deepen our understanding of how microbes present in the lower airways may affect initial events in the malignant transformation of airway epithelial cells, the immune surveillance needed to control nascent malignant cells, and the tumor's ability to proliferate and metastasize.

The complex cross-talk between dysbiotic lung microbiota and lung malignancies is an area of intense investigation 35. On one hand, certain members of the lung microbiome, primarily pathogenic species, have been associated with pro-inflammatory responses, creating a microenvironment conducive to cancer development. For instance, an increasing bacterial density with a particular abundance of *Streptococcus* and *Veillonella* species has been observed in patients suffering from lung cancer 36. These bacteria, through their metabolic by-products or direct interaction with the epithelial cells, appear to enable a pro-inflammatory milieu, fostering conditions that favor tumor progression. On the other hand, commensal bacteria in the pulmonary microbiota have suggested exerting protective effects against lung cancer. Various studies have proposed a potential role of commensals in modulating host immunity, maintaining pulmonary homeostasis, and directly inhibiting the growth of cancer cells 37. The mechanisms employed by these commensals to deter cancer growth are as yet unclear and demand exhaustive research.

The relationship between the pulmonary microbiota and the immune microenvironment draws fascinating insights into the complex interplay between microorganisms and host immunity. Increasing research highlights an intricate link between dysbiotic microbiota and higher cancer susceptibility 38. This association is mediated through a plethora of microbial-immune crosstalk mechanisms like chronic inflammation, immune suppression, and alterations in metabolic pathways 39.

Mechanistically, our results demonstrated that when the symbiotic bacteria in the lung are structurally abnormal, a large number of bacterial antigens continuously stimulate NK cells in the tumor microenvironment, resulting in the increased expression of the inhibitory receptor TIGIT on the surface of NK cells and CD155 on tumor cells. When the CD155 of tumor cells binds to the TIGIT of NK cells, the expression of CD155 on tumor cells increased, rendering the NK cells exhausted. Simultaneously, IL-2 and IFN-γ secretion is reduced, which further reduces NK cell proliferation and activation and ultimately promotes tumor proliferation. Unfortunately, we did not elucidate the molecular mechanisms underlying the formation of the immunosuppressive microenvironment mediated by the pulmonary microbiota. Therefore, it is imperative to further amalgamate *in vivo* and *in vitro* experiments in future endeavors to unravel how microorganisms modulate the heightened expression of TIGIT in NK cells and its underlying rationale. However, a systematic understanding of intricate interplay is warranted. Thus, the connection between pulmonary microbiota and the immune microenvironment warrants further exploration to harness microbiota-immunity crosstalk for potential therapeutic benefits.

## Supplementary Material

Supplementary figure.

## Figures and Tables

**Table 1:** Clinical characteristics of patients with lung cancer

Clinical characteristics	N0 (n=12)	N1-N3 (n=7)	P value
Age (years), mean ± SD	58.92 ± 5.63	63.23 ± 8.55	0.253
Gender			0.862
Female	5	3	-
Male	7	4	-
BMI (kg/m^2^), mean ± SD	25.68 ± 2.77	26.75 ± 3.52	0.963
Tumor diameters (cm)	1.59 ± 0.65	2.26 ± 0.87	0.035
Smoking history			0.663
Yes	7	5	-
No	5	2	-
Pathology			0.528
LUAD	12	7	-
LUSC	0	0	-
Family history			0.963
Yes	1	1	-
No	11	6	-

## Abbreviations

COPD: chronic obstructive pulmonary disease
HIV: human immunodeficiency virus
ERK: extracellular signal-regulated kinase
PI3K: phosphoinositide 3-kinase
SPF: specific pathogen-free
GF: germ-free
BALF: bronchoalveolar lavage fluid
OTU: operational taxonomic unit
PCoA: principal coordinates analysis
CCK8: Cell Counting Kit-8
TME: tumor microenvironment
IL-2: interleukin-2
IFN-γ: interferon-gamma
