# Supplementary material for: Lung commensal bacteria promote lung cancer progression through NK cell-mediated immunosuppressive microenvironment
Source: Int J Med Sci. 2025 Feb 3;22(5):1039–51. doi: 10.7150/ijms.107026 (PMC11866540; doi:10.7150/ijms.107026)
Supplement: Supplementary file 1 — Supplementary figure. [file ijmsv22p1039s1.pdf]

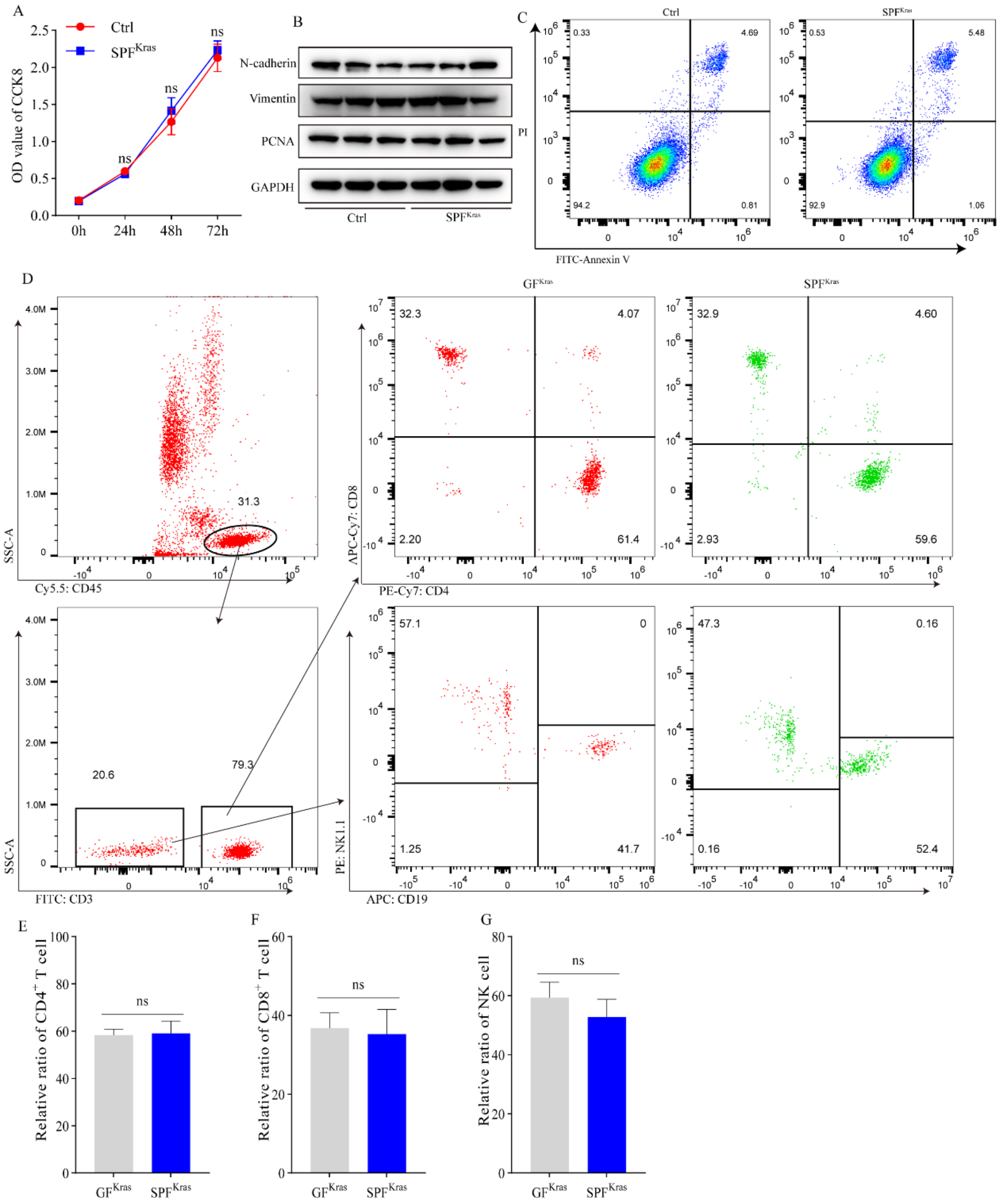

Fig S1, Microbiota promotes progression of lung cancer via TME. A-C Proliferation (A), migration (B) and apoptosis (C) of LLC was observed, but no significant difference was found between SPF^{Kras} and control. D-G There were no significant differences in $CD4^{+}$ T cells (D, E), $CD8^{+}$ T (D, F) and NK cells (D, G) between SPF^{Kras} and GF^{Kras} mice.
